# Perceptions of Barriers to and Facilitators of Participation in Health Research Among Transgender People

**DOI:** 10.1089/trgh.2016.0023

**Published:** 2016-10-01

**Authors:** Ashli A. Owen-Smith, Cory Woodyatt, R. Craig Sineath, Enid M. Hunkeler, La Tasha Barnwell, Ashley Graham, Rob Stephenson, Michael Goodman

**Affiliations:** ^1^ Department of Health Management and Policy, School of Public Health, Georgia State University, Atlanta, Georgia.; ^2^ Department of Epidemiology, Rollins School of Public Health, Emory University, Atlanta, Georgia.; ^3^ Division of Research, Kaiser Permanente Northern California, Oakland, California.; ^4^ Department of Behavioral Sciences and Health Education, Rollins School of Public Health, Emory University, Atlanta, Georgia.; ^5^ Department of Anthropology, University of Connecticut, Storrs, Connecticut.; ^6^ Department of Health Behavior and Biological Sciences, School of Nursing, University of Michigan, Ann Arbor, Michigan.

**Keywords:** content analysis/narrative analysis, focus group, gender identity, transgender

## Abstract

**Purpose:** Although transgender people may be at increased risk for a range of health problems, they have been the subject of relatively little health research. An important step toward expanding the evidence base is to understand and address the reasons for nonparticipation and dropout. The aim of this study was to explore the perceptions of barriers to and facilitators of participation in health research among a sample of transgender people in San Francisco, CA, and Atlanta, GA.

**Methods:** Twelve in-person focus groups (FGs) were conducted; six (three with transwomen, three with transmen) were conducted in San Francisco and six FGs were conducted in Atlanta (three with transwomen and three with transmen). FGs were audiorecorded, transcribed, and uploaded to MaxQDA software for analysis. A codebook was used to code transcripts; new codes were added iteratively as they arose. All transcripts were coded by at least 2 of the 4 researchers and, after each transcript was coded, the researchers met to discuss any discrepancies, which were resolved by consensus.

**Results:** Among 67 FG participants, 37 (55%) identified as transmen and 30 (45%) identified as transwomen. The average age of participants was ∼41 years (range 18–67) and the majority (61%) were non-Hispanic Whites. Several barriers that can hinder participation in health research were identified, including logistical concerns, issues related to mistrust, a lack of awareness about participation opportunities, and psychosocial/emotional concerns related to being “outed.” A broad range of facilitators were also identified, including the opportunity to gain knowledge, access medical services, and contribute to the transgender community.

**Conclusion:** These findings provide insights about the perceived barriers to and facilitators of research participation and offer some guidance for researchers in our ongoing effort to engage the transgender community in health research.

## Introduction

Although transgender people may be at increased risk for a range of physical and mental health problems^1^ they have been the subject of relatively little health research.^2^ This may, in part, be due to methodological difficulties that are common when conducting research with sexual and gender minority (SGM) populations, specifically transgender people.

First, gender identity is a multifaceted concept, and defining it operationally for research purposes can be challenging.^3^ Second, participants may be reluctant to answer questions about their sexual behavior and/or gender identity, perhaps because of mistrust of the research community.^4^ Third, because transgender people represent a relatively small proportion of the U.S. population, it is labor-intensive and costly to recruit a sample size adequate for rigorous statistical analysis.^1^ Finally, even for studies with sufficient staffing and funding, issues related to nonparticipation and dropout may severely impede recruitment and retention efforts.^5^ An important step toward expanding the evidence base on health issues among transgender people is to understand and address the reasons for research nonparticipation and dropout.

In part, because of the well-known challenges related to recruiting and retaining racial and ethnic minorities in clinical research, the available literature on barriers to and facilitators of research participation has largely focused on these populations. This literature suggests that there are a number of barriers to racial and ethnic minority research participation, including perceptions of discrimination, fear and mistrust, lack of access to or knowledge about research, and the perceived burden (e.g., time and financial constraints) associated with participation.^6–9^ By contrast, there are only two known studies to date that have specifically explored barriers to and/or facilitators of research participation among transgender people. One study on participation of transgender women in preventive HIV vaccine trials reported that stigma, mistrust of the scientific community, lack of exposure to information about trials, and concerns about vaccine side effects were commonly reported barriers to participation. By contrast, culturally competent staff, recommendations by a trusted provider, and assistance with basic needs were identified as facilitators to participation.^10^ The other study that explored research participation among HIV-positive transwomen reported that concerns about being exploited, dehumanized, and/or judged were significant barriers to participation; factors such as providing appropriate financial compensation, confidentiality, and the belief that participants could directly benefit from the research were reported as facilitators.^11^ Although these two studies provide meaningful insights about perceived barriers to and facilitators of research participation among transwomen, these findings may not be applicable to other types of studies enrolling transgender individuals—for example, studies involving HIV-negative individuals and/or transmen. Therefore, the aim of this study was to explore the perceptions of barriers to and facilitators of participation in health research among a sample of transgender men and women in San Francisco, CA, and Atlanta, GA. Identifying and addressing barriers, as well as understanding what motivates participation can aid researchers in their efforts to more effectively recruit and retain transgender individuals in health research going forward.

## Methods

### Study design

Focus groups (FGs) were used for data collection because this approach allows researchers to understand individuals’ interpretation of experiences, provides opportunities for in-depth probing of those experiences, and promotes community involvement in the research process,^12–14^ which was an important goal of this study. Furthermore, FGs can be used to explore a broad range of topics, with a wide variety of individuals, including participants with lower education levels who may be more difficult to reach through quantitative methods.^15^

### Procedures

Twelve in-person FGs were conducted. Six were conducted in San Francisco, three of which were with transwomen and three with transmen. Similarly, six FGs were conducted in Atlanta, three of which were with transwomen and three with transmen. Participants were recruited through a variety of methods: community outreach through social media, participant or healthcare provider referral, flyers, and an online message board. Interested individuals were given a study phone number and the link to the study website, which provided more information about the study and an opportunity to consent to being contacted by study staff through telephone. Study staff then contacted interested participants, described the study in greater detail, answered questions, and, if a person expressed the desire to participate, screened the individual for eligibility. Individuals were eligible to participate if they self-identified as a female-to-male or male-to-female transgender person and were 18 years of age or older. Eligible individuals were then scheduled for an FG discussion and compensated with a $40 gift card for their time. Each group comprised five to eight participants and was facilitated by two trained study staff members. The FGs lasted ∼2 h and were audiorecorded. The audiorecordings were then professionally transcribed verbatim and destroyed. The resulting transcripts were uploaded to MaxQDA software for analysis. The Institutional Review Boards of participating research centers approved the study.

### Data analysis

The FG guide consisted of semistructured, open-ended questions that addressed three domains: (1) perceived health and healthcare issues, (2) sources and perceived quality of information about health and healthcare issues, and (3) barriers to and facilitators of research participation. The data codebook outlining relevant themes across all topic areas, using the FG questions as initial categories, was used to guide the thematic analysis, which was conducted iteratively. A team of four researchers, each coded the first FG transcript independently and, met to discuss the emerging themes, identify congruence with and departures from the existing codebook, and refined the codebook accordingly. The revised codebook was then used to code subsequent transcripts and new codes were added as they arose. All transcripts were coded by at least two of the four researchers and, after each transcript was coded, the researchers met to discuss any discrepancies, which were resolved by consensus.

## Results

### Sociodemographic characteristics

Among 67 FG participants, 37 (55%) identified as transmen and 30 (45%) identified as transwomen. The average age of participants was ∼41 years (range 18–67) and the majority (61%) were non-Hispanic Whites. Most participants were recruited through peer or community member referrals (Table 1).

**Table 1.** **Focus Group Participants’ Demographics**

		By region	
Characteristics	Total (*N*=67)	San Francisco (*N*=35)	Atlanta (*N*=32)
Gender identity, *N* (%)			
Transmen	37 (55)	20 (57)	17 (53)
Transwomen	30 (45)	15 (43)	15 (47)
Mean age (SD)	40.8 (12.2)	40.7 (12.7)	41.0 (11.9)
Race/Ethnicity, *N* (%)			
Non-Hispanic White	41 (61)	24 (69)	17 (53)
African American	22 (33)	7 (20)	15 (47)
Asian	2 (3)	2 (6)	0 (0)
Hispanic/Latino	1 (1.5)	1 (3)	0 (0)
Other	1 (1.5)	1 (3)	0 (0)
Recruitment method, *N* (%)^a^			
Referral	26 (39)	13 (37)	15 (47)
Social media	13 (19)	0 (0)	13 (41)
Flyer/newsletter	11 (16)	9 (26)	2 (6)
Message board	6 (9)	6 (17)	0 (0)
Other	5 (7)	3 (9)	2 (6)
Unknown	7 (10)	7 (20)	0 (0)

% may not sum to 100 due to rounding.

^a^ *N* and % do not sum to 100 as responses are not mutually exclusive.

### Barriers to research participation

The FG discussions revealed a broad range of barriers that can hinder participation in health research, including logistical concerns/challenges, issues related to mistrust, a lack of awareness about participation opportunities, psychosocial/emotional concerns related to being “outed” as a result of participating, and financial concerns.

#### Logistical concerns

Participants in all 12 FGs consistently reported that logistical issues were a significant barrier to participating in health research. For example, participants revealed that many individuals in the transgender community do not own cars or have access to private transportation and thus might find it difficult to travel to a research site. This is particularly true for those who live in more suburban or rural areas. Many participants felt that many transgender individuals are financially disadvantaged, often working multiple jobs and/or frequently moving for employment. For this reason, they may not have the time/ability to participate in research. Similarly, participants indicated that some members of the transgender community do not have personal cell phones or computer access, therefore contacting and returning phone and/or email messages from research staff in a timely manner are often difficult.

#### Lack of trust

Across all FGs, individuals indicated that a lack of trust in research and researchers is a barrier to participation. Participants questioned researchers’ motives and expressed frustration that neither they nor their community typically benefit from participation in a meaningful way. According to one participant:

There is just a historical hesitancy for trans people to get involved in research because, ‘Who's doing it? What are they doing it for? Where's the information going?’ Too often … it doesn't get shared back with us. People will show up at conferences, get all of our information, and then we never hear the results of their studies.

Participants also felt that the researchers, just like others encountered outside of a research context, might behave in judgmental or discriminatory ways. One individual stated:

I think trans people in general just distrust because we're either being quickly judged or prejudiced against or put into a category right away. So we're always just like, “What are they thinking? What are they trying to get from us? What kind of perverts are they this time?

#### Lack of awareness of research

Participants across all of the FGs expressed concern that transgender individuals who do not have access to the Internet and/or are not accessing healthcare or social services may not be aware of research participation opportunities. Although participants felt that many transgender individuals might be willing to participate, as one participant stated, “you can't show up for something you don't know about.”

#### Psychosocial/emotional concerns

Participants consistently expressed concerns that participating in research might “out” them and consequently jeopardize their personal and/or professional relationships and safety. One individual stated:

I think that most people are scared like what's just around them when they get there—is there going to be a big sign that says something about ‘transgender people this is where you come’, or is everybody in the building gonna know that everybody that's going in that room is transgender. So that might be a fear of why some people are not coming, because they're thinking, does anybody else know what's going on in that room?

Individuals reported feeling fearful of the possible consequences of participating in research and indicated that this was a significant barrier to participation.

#### Financial concerns

Although most individuals appreciated receiving a financial incentive for their participation, some felt that $40 was not sufficient for a 2-h FG. This was particularly true for those individuals who had to pay for public transportation to the research site, because their transportation costs would be deducted from their research compensation (“you are paying me $40 but it takes me $5 to get here so really you're paying me $35”). Participants also expressed dissatisfaction with receiving a gift card rather than a cash incentive. One individual stated:

I was interested when I heard it was going to be $40, but when I heard it was going to be a gift certificate and we were restricted to where we were going to use that...if I hadn't already signed up, I probably wouldn't have in the first place if I knew it was a gift card. Because … when you're dealing with so much money problems in your life … it is incredibly valuable to get any money incoming.

### Facilitators of research participation

The FGs also revealed a broad range of facilitators that may increase transgender individuals’ participation in health research. These facilitators involve logistical factors, incentives, the opportunity to gain knowledge and receive medical services, the ability to contribute to their community, the involvement of transgender research staff, perceived credibility, researcher transparency, and the use of various promotion and recruitment methods.

#### Logistical facilitators

Respondents felt that some logistical factors might increase the likelihood of participation in future research. For example, several individuals indicated that they would be more likely to participate if the research site had free parking or was close to public transportation, if there were was flexibility in scheduling participation (e.g., both weekday and weekend opportunities), and if there were options for how participation could occur (e.g., in person, by phone, by mail, and/or via the Internet).

#### Incentives

Some individuals reported being willing to participate without any financial incentive, to contribute to the transgender community. However, many indicated a need for incentives, stating that, at a minimum, food should be provided. Participants felt that this might be particularly important for transwomen who, according to several participants, “are working girls and may not have eaten” (many respondents indicated that some of their male-to-female transgender peers are sex workers and often report experiences of homelessness and food insecurity). Having options for the type of incentive was seen as desirable by many, as some individuals preferred cash, while others preferred public transportation cards or the ability to donate the earned money to a charity of his/her choice. Most individuals agreed that they would be most likely to participate in future studies if researchers covered the cost of transportation to the study site (in the form of a bus/train voucher), gave a small financial incentive (ideally in the form of cash), and provided food during the study session.

#### Gaining knowledge

Many individuals reported that they were motivated to participate in research to stay informed about current scientific developments in transgender health (e.g., to “get some type of information about what's going on in the studies”). Another reason to participate was the opportunity to network with others in their community in the event that they might gain some new information about healthcare services/providers, employment opportunities, social services, or other desired resources. For example, one participant stated:

You know we're all sitting here right here, right now, because we're trying to find resources anywhere we can, the best way we can.

Relatedly, several individuals indicated that they would be more likely to participate if they would receive updated lists of healthcare and social service resources (providers?), known to be transfriendly.

#### Gaining medical services

Across all FGs, individuals indicated that receiving medical services as part of the study would increase their likelihood of participation; this was seen as particularly important for many in the transgender community who cannot afford regular healthcare. For example, one participant stated:

You know, you're talking about a group that has a lot of medical needs that they may not have access to. And if there is some way to incentivize people by offering them some of the medical care that they're lacking in some way, particularly the people that are difficult to reach, the people who really need to be a part of these studies. I've had lapses in insurance since I started hormones. But I've been pretty fortunate overall with being able to not have much lapse in taking hormones, and in my medical care. I think there are a lot of people out there that aren't as fortunate, that for an incentive that would somehow involve them being able to access the care that they need, that would be huge.

#### Contributing to the community

Many individuals in each of the FGs reported that they felt motivated to participate in studies because they wanted to contribute to research that they hope will benefit their community. Some individuals commented on the lack of information on mental health issues in the community or the long-term effects of hormonal therapies on health outcomes, and felt that they could provide data that might help to address these knowledge gaps. Others felt that their participation might be one way to share information with the scientific community as well. One participant stated:

I want to participate because we need this research and because it seems like there is finally somebody willing to do it. I mean, when I transitioned, nobody was doing any research. So I think just the fact that there are people that want to do it, like, makes me want to participate so we can get the information.

#### Transgender or transfriendly research staff

Participants across all groups reported that they would be more likely to participate in research if the study recruitment and/or data collection were conducted by a transgender person. For example, one individual felt that study staff who did not identify as transgender would “not understand who I am, where I'm coming from, who's in my community, what the variations are within my community, and so forth.” Similarly, another participant suggested that,

Even if you can't get transgender people to completely run the entire operation, but if you can keep the transcommunity deeply involved in the process, then you're much more likely to get people to come.

Most participants felt that, if it were not possible to hire a transgender individual for study recruitment and/or data collection, study staff should have a basic understanding of transgender issues and be trained to use culturally competent, appropriate language. As one participant stated,

So I think making sure that all of your researchers read a variety of trans narratives from the first person perspective can help, so you know that way we can be assured that y'all have picked up on various languages, various nuances, you know vocabulary that you should be using or should be avoiding, and stuff like that. It would make us feel more comfortable.

Several individuals reported that, while participating in prior studies, they did not feel that study staff had adequate training (e.g., a research assistant did not know the correct terminology for some common gender confirmation surgeries) and that they had been treated inappropriately by study staff (e.g., a staff person had used an incorrect pronoun). These participants acknowledged that these types of experiences made them hesitant to participate in future studies.

#### Perceived credibility

Participants across all FGs noted the importance that the overall study be associated with a reputable institution. For example, one individual reported that he/she would participate in a study, “as long as it was somewhere that was academic and respectable.” Another respondent also commented that one of the research sites for this study needed to be further emphasized in recruitment materials because, “I think just putting it out there that it's being held at a medical center, because that puts validation to the research.” In general, most participants agreed that the affiliation of the researchers was critical and, specifically, “who the source is, are they reliable, are they a known source, [and] are they affiliated with any other trustworthy sources” were all factors that could increase their likelihood to participate.

#### Study transparency

Many FG participants shared that they have consented to participate in previous studies but, at that time, did not understand the purpose of the research. One participant suggested that researchers need to be “more upfront about what you're doing because that increases the level of comfort, makes us feel … less objectified. and like I'm being a guinea pig.” Furthermore, several individuals reported that they would be more likely to participate in research if the benefit of that research to their community was communicated. As one individual stated:

I guess it's also helpful to, we were able to understand, for the most part, why we were doing this … if you could give us like a head's up on where's this going, what is this going to do, how is this going to help anybody. Like what happens after, what kind of change it causes, then if we could hear some of that it would probably help greatly.

#### Study promotion and recruitment

Individuals reported that their willingness to participate in research was, in part, dependent on how and where they were recruited. As described earlier, recruitment by transgender or transfriendly staff was seen as important and so, not surprisingly, participants indicated that word of mouth from members in the transgender community would likely be the most effective recruitment method. In part, because of the credibility that these institutions possess, LGBT-friendly churches, community and social service organizations, and community-based businesses (such as bookstores and coffee shops) were also suggested for recruitment venues. Many of the FG participants reported spending a lot of time on social media and on transgender blogs and listservs and thus felt that these are reliable methods for reaching some transgender individuals. However, several individuals acknowledged that researchers would likely miss important subgroups in the transgender community using an Internet-based approach, such as those who are homeless or otherwise economically disadvantaged. Therefore, several participants suggested that researchers should also consider recruiting at homeless shelters and community-based health clinics, and through support groups. Finally, festivals (such as Pride) and conferences (such as The Southern Comfort Conference or the Philadelphia Trans-Health Conference) were mentioned as other forums that might be perceived as credible and allow researchers to recruit a more diverse group of transgender community members.

## Discussion

Although the body of literature related to transgender health is growing, much of the available evidence to date is limited because of small sample sizes, the use of self-report, and cross-sectional or retrospective study designs.^5,16,17^ Important gaps in the scientific evidence include the following: healthcare access and utilization patterns over time, determinants of hormonal and surgical treatment complications, and rates of chronic age-related conditions that might be related to hormonal therapy. Closing these gaps will require identifying, recruiting, and following diverse cohorts of transgender people.^5^ Furthermore, an evaluation of health system interventions aimed at addressing barriers to transgender individuals receiving high quality care to improve transgender health is desperately needed.^1,17^ These types of studies will similarly require effective enrollment and retention of transgender participants. Consequently, it is critical to carefully examine the factors that might discourage or motivate transgender individuals’ participation in both longitudinal cohorts and intervention studies. This study found that, although there are several significant barriers to research participation for this population, myriad facilitators were identified in these discussions, revealing a multitude of helpful insights for researchers in the future, who might be planning such studies.

In general, many of the barriers to and facilitators of research participation reported by our transgender FG participants were similar to those reported in other studies, particularly those with racial/ethnic minorities and other populations that are adversely affected by health disparities. For example, logistical and financial issues were the most commonly reported barriers to research participation. Transportation-related challenges impeded individuals’ ability to travel to the research site and issues related to telephone and Internet access prevented contact and/or follow-up with research staff. This finding is consistent with prior literature focused on barriers to research participation for other underserved minority groups. One recent review of barriers to and facilitators of participation of minorities in clinical trials identified the lack of transportation to and from the research site, as well as the travel distance required, as significant barriers to research participation.^9^ These and other accessibility-related barriers mentioned in previous studies (e.g., not having a telephone, computer, or Internet access,^18^ interference with work or job responsibilities,^18^ lack of time,^19^ inconvenience,^20^ and difficulty scheduling appointments due to lack of flexibility on the part of study personnel^21^) present a significant impact on likelihood of participation, particularly among racial/ethnic minorities or individuals of a lower socioeconomic status. It is not surprising, therefore, that individuals in this study reported that, the amount and type of financial incentives offered to participants can act as an additional barrier to participation if these incentives are not perceived to be sufficient with respect to offsetting the logistical and financial cost of participation.

In this study, participants overwhelmingly indicated that these types of logistical and financial barriers, although significant, could be ameliorated if researchers (1) made participation more convenient (e.g., provided weekday and weekend opportunities to participate both during the day and in the evening, and conducted the research at a location near public transportation) and (2) offered incentives that acknowledged their needs and preferences (e.g., provided reimbursements for time and transportation costs, food, educational resources, and/or healthcare services). These types of facilitators were similarly reported in a recent systematic review conducted by George et al. that focused on research participation among racial/ethnic minorities.^7^ In this review, the most commonly articulated facilitators to health research participation across the included studies were those related to the perceived benefits to participation, such as receiving adequate remuneration (e.g., transportation provisions,^22^ monetary incentives,^23^ and a free lunch^22^) and access to healthcare resources (e.g., free health examinations/clinical services^24–26^). In both this review and in our study, potential participants reported that they desire incentives in the form of direct healthcare and/or informational resources, which might be otherwise unavailable to them.^7^

In the George et al. systematic review, a lack of access to information/awareness about research opportunities was a barrier that was reported across all racial/ethnic minority groups and was represented in almost one third of the articles included in the review.^7^ Andrasik et al. also reported that the lack of exposure to information was the most frequently cited barrier to HIV vaccine trail participation among transwomen.^10^ We similarly found that many of the participants were concerned that, although *they* had been made aware of the FG opportunity, researchers were unlikely to reach some important subgroups within their community—for example, those who have not yet “come out” as transgender, those who are commonly referred to as “stealth” (individuals who, after beginning transition and living in their preferred gender, do not readily reveal their birth-assigned gender), individuals who are sex workers, and those who are homeless. Biernacki and Waldorf have articulated the challenges of reaching these types of subgroups and argue that these populations, “because of moral, legal, or social sensitivities … have a very low visibility and as a result, pose some serious problems for location and contacting potential respondents.”^27^ The particular challenges associated with recruiting those who have not yet come out or who are “stealth” represent a unique barrier articulated by our FG participants and one not previously reported in the literature.

Participants in our FGs offered some insight as to how these challenges might be addressed (i.e., how to facilitate participation) by expanding the venues in and mechanisms by which potential participants are recruited. For example, respondents emphasized the importance of disseminating information about the study using a variety of methods, including those that are Internet based, by mail, and in-person, and, for those that are in-person, recruiting at a variety of locations, including conferences, festivals, community health centers/social service organizations, and other local businesses, and support groups. Prior evidence supports the notion that, particularly when conducting community-based recruitment, some combination of multiple approaches is essential, as a single-strategy approach is ineffective.^28^

Mistrust of the medical and research community, concerns about privacy, and issues related to stigma were also commonly reported as significant barriers to research participation in our FGs. Many transgender individuals in this study reported that they often did not fully understand the aims of the studies in which they participated and doubted whether the study results would ultimately have any impact on their community. These concerns have been described frequently in prior research, particularly among racial/ethnic minorities. For example, findings from the George et al. systematic review indicate that mistrust was a reported barrier to participation across all four racial/ethnic minority groups and appeared in 77.3% of all articles included in the review.^7^ In some cases, this mistrust was related to a fear of purposeful mistreatment, whereby participants were worried about being treated like “laboratory rats” or “guinea pigs”; this concern was shared by many of our participants as well. Several participants in a recent qualitative study with HIV-positive transwomen in Canada similarly reported that, when they participated in research, they often felt exploited (“we're their science project”) and dehumanized (“we're basically rats to them”).^11^ In other cases, this mistrust was associated with the belief that the researcher's agenda did not serve the community or was not in the community's best interest; this issue was also raised by our participants. Transwomen in the Andrasik et al. study similarly expressed suspicion about the motives of researchers, in part, because of prior experiences with discrimination by healthcare professionals.^10^

FG participants in this study also worried that participation might result in the unwanted disclosure of their transgender status and that they might be judged, stigmatized, or misunderstood by the research staff. These concerns about being “outed” as a result of participation represent an important, unique barrier not reported in previous studies that focused on racial/ethnic minorities; however, more generalized fears associated with a perceived loss of privacy or lack of confidentiality resulting from research participation have been previously identified.^29^

Although our FG participants noted that barriers related to mistrust, privacy, and stigma were widespread and deeply rooted in the transgender community, they also articulated several important facilitators for participation that could serve to counteract these barriers. One, the presence of transgender staff was seen as an important way to build trust and demonstrate cultural sensitivity on the part of the research team. This finding is consistent with prior research, which suggests that pairing culturally congruent participants and recruiters plays an important role in increasing research participation,^30,31^ in part, because this gives research a “personal touch”^32^ and participants may prefer research staff whom they can relate to and communicate with in “their own language and rhythm of expression.”^24,33^ Transwomen in the Andrasik et al. study similarly expressed the importance that study outreach be conducted by culturally competent staff and/or facilitated by trusted transfriendly healthcare providers.^10^ Two, participants indicated that they would be more likely to participate if researchers were transparent about the purpose of the research and clear about the ways in which the study results might have a meaningful impact on the transgender community. Other previous studies have similarly reported that individuals are more likely to participate in research studies if they receive adequate information regarding the purpose and benefits of the study^19^ and if they believe that their participation will help others and/or benefit their community in the present and future.^7,9^ In some cases, this sense of altruism may even supersede other initial motivations to participate in research.^34^

### Recommendations

As noted by Waheed et al., it is important for researchers to plan strategies to overcome potential barriers to recruitment early in the research process (as early as the proposal writing stage), as these can be labor- and cost-intensive efforts, and providing additional resources after the project has begun is often difficult.^35^ We offer several recommendations based on the findings from this study to inform future recruitment efforts for research with transgender populations.

One, researchers should include a variety of recruitment methods so as to maximize opportunities to identify harder-to-reach subgroups within the transgender community (e.g., homeless/low income individuals, those without Internet and/or consistent telephone access, and individuals of different ages and at different stages of transitioning). These methods might include a combination of Internet-based approaches (e.g., recruiting through social media and/or email list serves) and in-person approaches (e.g., recruiting at local businesses and events as well as through organizations perceived as credible by the community).

Two, researchers should attempt to involve transgender research staff in the recruitment process if possible and, if not feasible, deliver cultural competency training to any staff involved in recruitment and data collection processes.

Three, researchers need to be mindful about how the study is advertised/promoted, both during the recruitment phase and throughout data collection activities so that individuals are not “outed” as a result of expressing interest in and/or participating in the research. For example, posted signage directing individuals to the research site should be clear to participants, but appear generic for those not affiliated with the study.

Four, researchers should focus on maximizing accessibility for potential participants by holding research sessions at easily reached locations (e.g., near public transportation) and offering individuals convenient times (e.g., weekday and weekend options, both during the day and in the evening).

Five, incentives should ideally include free transportation to and from the research site or participants should be offered vouchers to cover the cost of transportation; individuals should also be compensated for their time, ideally in the form of cash at the time of participation, and provided food while participating, if appropriate. Additional incentives could include the provision of certain clinical services free of charge, and up-to-date lists of transfriendly providers and other community resources that might be of interest to participants.

Six, researchers should be transparent and thorough when explaining the purpose of the research and highlight the ways in which individuals’ participation will advance the science and improve physical and/or mental health outcomes of importance to the community. Researchers should also prioritize the return of research findings to participants, at the conclusion of the study, to further facilitate trust.

## Conclusion

Emerging evidence suggests that SGM in general, and transgender people in particular, face myriad health disparities^36^ and yet our understanding of these disparities—as well as how to address and overcome them—remains limited.^1^ To address these limitations and expand the evidence base, it is essential to successfully recruit, enroll, and retain transgender individuals in health research, which necessitates a comprehensive understanding of the reasons that may motivate or deter individuals from participating in research. This study aimed to explore these factors and provide specific recommendations for researchers who hope to effectively engage transgender individuals in health research in the future.

The data are limited to transgender individuals who participated in an FG in two, large metropolitan areas of the United States. Therefore, these findings are not necessarily generalizable to the broader transgender community, racial/ethnic minority populations, those who live in more suburban or rural areas, or those who do not subscribe to conventional gender distinctions and/or do not identify as transgender (e.g., individuals who identify as “genderqueer”). However, as noted by Kidd and Parshall, confidence in FG findings is increased when conducting multiple groups in multiple sites,^37^ which was done in this study. We believe that this enhances the integrity of the results, particularly because similar themes emerged with similar frequencies across the two geographically, socioculturally, and politically different sites, and across both male-to-female and female-to-male groups. Although we systematically evaluated intercoder reliability, the labeling of emerging themes and judgments about the importance and significance of these themes are a subjective process and, while all efforts were made to describe participants’ perspectives with accuracy and transparency, this work is fundamentally interpretative and influenced by the authors’ own perspectives and experiences.

In summary, these findings provide insights about the perceived barriers to and facilitators of research participation and offer some guidance for researchers in our ongoing effort to engage the transgender community in health research. Several published studies evaluated the effectiveness of various recruitment strategies, but none to our knowledge have specifically explored the effectiveness of different strategies among transgender participants. Therefore, additional studies are needed to test these strategies using experimental or quasi-experimental methods so that researchers and funding agencies can make informed decisions about how best to recruit and retain SGM individuals in research studies.^35^ Additional studies are also needed to specifically examine whether certain strategies are more or less effective for certain subgroups within the transgender population. For example, a certain recruitment or retention strategy might be effective for transmen, but not for transwomen, or a strategy might work well in the Southeastern United States, but work less well in other regions of the country. Finally, additional funding for research related to transgender health and healthcare is needed, and the level of this funding needs to reflect the costs associated with increasing research participation identified by our participants—namely, sufficient funds for participant travel reimbursement, cash incentives, food, and medical services. Unfortunately, a recent analysis of the proportion of studies funded by the National Institutes of Health that focused on SGM populations revealed that there has been insufficient funding to support strong empirical research on transgender health, particularly related to concerns other than HIV/AIDS.^38^ Increasing the number of funding opportunities—as well as increasing the level of funding for those opportunities—is an important way to advance knowledge and, consequently, health equity for SGM populations.

## Abbreviations Used

FG: focus group
SGM: sexual and gender minority
